# Expression optimization of recombinant cholesterol oxidase in *Escherichia coli* and its purification and characterization

**DOI:** 10.1186/s13568-018-0711-3

**Published:** 2018-11-12

**Authors:** Aliakbar Fazaeli, Abolfazl Golestani, Mostafa Lakzaei, Samaneh Sadat Rasi Varaei, Mahdi Aminian

**Affiliations:** 1 Department of Clinical Biochemistry, School of Medicine, Tehran University of Medical Sciences, Tehran, Iran; 2 Recombinant Vaccine Research Center, Tehran University of Medical Sciences, Tehran, Iran

**Keywords:** Affinity chromatography, Cholesterol oxidase, Expression optimization, Recombinant enzyme

## Abstract

Cholesterol oxidase is a bacterial flavoenzyme which catalyzes oxidation and isomerization of cholesterol. This enzyme has a great commercial value because of its wide applications in cholesterol analysis of clinical samples, synthesis of steroid-derived drugs, food industries, and potentially insecticidal activity. Accordingly, development of an efficient protocol for overexpression of cholesterol oxidase can be very valuable and beneficial. In this study, expression optimization of cholesterol oxidase from *Streptomyces* sp. SA-COO was investigated in *Escherichia coli* host strains. Various parameters that may influence the yield of a recombinant enzyme were evaluated individually. The optimal host strain, culture media, induction time, Isopropyl ß-d-1-thiogalactopyranoside concentration, as well as post-induction incubation time and temperature were determined in a shaking flask mode. Applying the optimized protocol, the production of recombinant cholesterol oxidase was significantly enhanced from 3.2 to 158 U/L. Under the optimized condition, the enzyme was produced on a large-scale, and highly expressed cholesterol oxidase was purified from cell lysate by column nickel affinity chromatography. K_m_ and V_max_ values of the purified enzyme for cholesterol were estimated using Lineweaver–Burk plot. Further, the optimum pH and optimum temperature for the enzyme activity were also determined. We report a straightforward and easy protocol for cholesterol oxidase production which can be performed in any laboratory.

## Introduction

Cholesterol oxidases (EC 1.1.3.6) are bifunctional bacterial flavoenzymes belonging to the family of oxidoreductase which catalyze the first step in the catabolism of cholesterol. They catalyze oxidation as well as isomerization of cholesterol and produce equimolar amounts of cholest-4-en-3-one coupled with hydrogen peroxide as the final products (Moradpour and Ghasemian 2016). There are two types of cholesterol oxidase (ChO) depending on the nature of the bond between FAD cofactor and apoenzyme. In type I, the FAD cofactor is linked to the protein through a noncovalent bond, while in type II, the cofactor is covalently bond to the apoenzyme (Vrielink and Ghisla 2009). Both types of enzymes have found wide applications as a useful biotechnological tool.

Cholesterol oxidase is the second most widely used enzyme in clinical laboratories (Doukyu et al. 2009). This enzyme is commonly used for determining cholesterol levels both in serum and in other biological samples (MacLachlan et al. 2000). On the other hand, the ability of cholesterol oxidase in bioconversion of 3β-hydroxysteroids makes it a valuable enzyme for transformation of sterols and non-sterols in the pharmaceutical industry (Doukyu 2009). Recently, many attempts have been made to reduce cholesterol levels in foods. The reduction of food cholesterol levels may occur via enzymatic methods (Yehia et al. 2015). Many experiments have been conducted to reduce milk and yolk cholesterol levels using cholesterol oxidase (Lv et al. 2002; Serajzadeh and Alemzadeh 2010; Smith et al. 1991). In addition, other investigations have addressed the role of cholesterol oxidase as an approach to pest control strategies (Cho et al. 1995; Purcell et al. 1993).

ChO has no mammalian homolog and is totally produced by pathogenic and nonpathogenic bacteria. Pathogenic bacteria employ this enzyme for infection of host macrophages by oxidation of membrane cholesterol, while nonpathogenic bacteria tend to utilize ChO as a metabolic tool for obtaining carbon sources from cholesterol decomposition (Pollegioni et al. 2009). So far, many efforts have been made to obtain the ChO from original microorganisms. Nevertheless, this approach suffers from some challenges such as difficult growth conditions and low productivity of original microorganisms (MacLachlan et al. 2000). In order to find a solution for these issues, ChO genes from different bacterial sources have been cloned and expressed which would be effective for commercial application of enzyme production (Brigidi et al. 1993; Corbin et al. 1994; Fujishiro et al. 1990; Horii et al. 1990; Liu et al. 1988; Molnár et al. 1991; Murooka et al. 1986; Nishiya et al. 1997; Ohta et al. 1992; Purcell et al. 1993; Solaiman and Somkuti 1991, 1995; Solaiman et al. 1992; Somkuti et al. 1991, 1995; Somkuti and Solaiman 1997). ChO from *Streptomyces* sp. SA-COO (ChOA) secretory production has been proved in a *Streptomyces* host-vector system (Murooka et al. 1986). Also, the *ChOA* gene has been cloned and sequenced (Ishizaki et al. 1989). Nomura et al. successfully expressed the *ChOA* gene in *Escherichia coli* (Nomura et al. 1995). Further, the thermal stability of the ChOA was improved in another study (Nishiya et al. 1997).

Recombinant ChOA production in a large quantity facilitates its biochemical characterization and its use in industrial processes. To this end, in the current study, we have taken a straightforward and effective approach to maximize ChOA production by optimizing the culture and induction parameters in shaking flasks.

## Materials and methods

### Strains, materials, and culture media

*Escherichia coli* host strains *BL21(DE3)*, *BL21(DE3)pLysS*, *and Rosetta*-*gami2(DE3)* were obtained from Novagen (Madison, WI, USA). Synthesis of plasmid pET24b-*ChOA* was ordered to Bio Basic Inc. (ON, Canada). Ni-CAM HC Resin, isopropyl-β-d-thiogalactopyranoside (IPTG), kanamycin and chloramphenicol were purchased from Sigma-Aldrich (MO, USA). All other chemicals were prepared from Merck chemical company (Darmstadt, Germany). The following liquid media were used: Luria–Bertani (LB, 10 g/L peptone, 5 g/L yeast extract, 5 g/L NaCl, Merck), Super Broth (SB, 32 g/L peptone, 20 g/L yeast extract and 5 g/L NaCl, Merck), Terrific Broth (TB, 12 g/L peptone, 24 g/L yeast extract, 8 g/L glycerol, 17 mM KH_2_PO_4_ and 72 mM K_2_HPO_4_, Merck).

### Optimization of recombinant ChOA expression

#### Expression of ChOA in different *E. coli* hosts

Initially, three different *E. coli* strains capability for the production of recombinant ChOA were assessed under our routine laboratory conditions. At first, *ChoA* gene (GenBank accession number M31939) was designed into pET24b(+) expression plasmid between *Nde*I-*Bam*HI restriction sites (GenBank accession number MH810339). Then, 1 µL of pET24-*ChOA* plasmid was transformed into chemically competent cells of *BL21(DE3)*, *BL21(DE3)pLysS*, and *Rosetta*-*gami2(DE3)* host strains. We used 50 µg/mL kanamycin in the solid and liquid medium of each of the three strains and additional 25 µg/mL chloramphenicol in the case of *BL21(DE3)pLysS* and *Rosetta*-*gami2(DE3)*. After overnight incubation, a single colony of each strain was taken from LB agar plates and used for inoculation of 3 mL pre-culture media and incubated at 37 °C, 160 rpm for 12 h. On the following day, 10 mL of LB media was inoculated by 100 µL of pre-culture media and incubated under the same conditions. When the optical density at 600 nm (OD_600nm_) reached 0.6, IPTG was added up to a final concentration of 0.5 mM. The cells were harvested after 6 h by centrifugation at 7000×*g*, 4 °C, and within 10 min. The harvested cells were resuspended in 0.5 mL of PBS buffer containing NaCl (0.3 M) at pH 7. Bacterial cells were disrupted by sonication and the lysate was centrifuged at 13,000×*g*, 4 °C, within 20 min. The productivity of each host strain was evaluated by enzyme activity assay in the crude extract. ChOA activity was measured at 25 °C by a modification of the method of Allain et al. (1974) and Doukyu et al. (2008). The assay mixture contained 100 mM potassium phosphate pH 7.0, 1 mM cholesterol, 21 mM phenol, 1.4 mM 4-aminoantipyrine and 5 U/mL peroxidase. The reaction was started by addition of 100 µL sample to 1 mL assay mixture and the appearance of the red chromophore was monitored continuously at 500 nm. Blanks without enzyme or without cholesterol were routinely run in parallel. One unit of activity was defined as the formation of 1 µmol of hydrogen peroxide (0.5 µmol of quinoneimine dye) per min at 25 °C.

#### Culture media optimization

To determine the optimal culture media, the overnight culture of *BL21(DE3)pLysS* harboring pET24-*ChOA* plasmid was made in 3 mL of LB media. Then, 10 mL of three different medium types including LB, TB, and SB were inoculated with a pre-culture with the ratio of 1:100. When OD_600nm_ reached 0.6, the cultures were induced with 0.5 mM IPTG and incubated at 37 °C, 160 rpm for 6 h. The cultures were harvested and the pellet was resuspended in 0.5 mL of PBS buffer. After sonication, the cell lysate was centrifuged at 13,000×*g*, 4 °C, for 20 min. The total activity of recombinant ChOA was measured by performing enzyme assay in the supernatant crude extract to determine productivity.

#### Optimum induction time

*BL21(DE3)pLysS* cells containing pET24-*ChOA* were grown overnight in LB media. Fresh culture (4 flasks) containing 10 mL TB media was inoculated (1:100) and incubated at 37 °C, 160 rpm. When the OD_600nm_ of cultures reached 0.3, 0.6, 1.2 and 1.8, induction was made with 0.5 mM IPTG. Each culture was incubated for 6 h at 37 °C, 160 rpm. The harvested cells were resuspended in 0.5 mL of buffer (PBS, pH 7) and disrupted by sonication, then centrifuged at 13,000×*g*, 4 °C, for 20 min. Quantification of active (soluble) enzyme was performed by enzyme activity assay.

#### Optimum IPTG concentration

The effects of various IPTG concentrations on ChOA productivity were further evaluated. For this purpose, five flasks containing 10 mL of TB media were inoculated by a pre-culture with the ratio of 1:100. The cultures were incubated at 37 °C, 160 rpm until OD_600nm_ reached 0.6. The cell cultures were induced by IPTG concentrations of 0.05, 0.1, 0.25, 0.5, and 1 mM respectively. After disruption and centrifugation of harvested cells, enzyme expression was measured by enzyme activity assay.

#### Induction temperature and post-induction incubation time

The productivity of recombinant ChOA was evaluated at different incubation temperatures (15 °C, 25 °C, and 37 °C), as well as four different post-induction incubation times (6, 8, 16, and 24 h). These parameters were investigated in three flasks containing 20 mL of TB media, inoculated by 0.2 mL of pre-cultured *BL21(DE3)pLysS* harboring *ChOA* gene. The induction was done at OD_600nm_ ≃ 0.6 by adding IPTG in a final concentration of 0.25 mM. After the induction, the flasks were incubated at 15 °C, 25 °C, and 37 °C on a rotary shaker with a speed of 160 rpm. In order to determine the optimal post-induction incubation time, 2 mL of culture media from each flask was withdrawn at different time (6, 8, 16, and 24 h) intervals. The collected samples were centrifuged and pellets were resuspended in the buffer, and then the cells were disrupted by sonication. Once the samples were prepared, enzyme activity assay performed for quantification of the expressed recombinant enzyme.

### Large-scale expression of ChOA under optimized condition

Overexpression of *ChOA* gene was performed according to the results of optimized protocol. A pre-culture was made by inoculating 5 mL of LB media containing kanamycin (50 µg/mL) and chloramphenicol (25 µg/mL) with pET24-*ChOA* harboring *BL21(DE3)pLysS* cells. Then, 500 mL of TB media containing 50 µg/mL kanamycin and 25 µg/mL chloramphenicol was inoculated by the pre-culture. When OD_600nm_ reached 0.6, induction of *ChOA* gene expression was done by adding IPTG up to a final concentration of 0.25 mM and continued with 24 h incubation at 15 °C, 160 rpm. The harvested bacterial pellet was resuspended in 10 mL of buffer (PBS, NaCl 0.3 M, and Imidazole 5 mM, pH 7) and disrupted by sonication. The cell lysate was centrifuged at 13,000×*g*, 4 °C, for 20 min and the supernatant used for ChOA purification via affinity chromatography.

### Purification of recombinant ChOA

Recombinant ChOA containing N-terminal His tag was purified from the soluble crude extract using nickel affinity chromatography (Ni-CAM HC Resin). The column (2 mL) was equilibrated with 30 mL of equilibration buffer (PBS, Imidazole 5 mM, NaCl 0.3 M; pH 7) at 1 mL/min. The supernatant was loaded onto the column and the column was washed with equilibrium buffer until the absorbance at 280 nm reached the basal level. To elute the protein, elution buffer (PBS, NaCl 0.3 M, and Imidazole 200 mM; pH 7) was used, and the released proteins were fractionated. The purity of the fractionated samples was evaluated by SDS-PAGE 12%. The pure fractions were pooled together and dialyzed against 50 mM sodium phosphate buffer at 4 °C, pH 7 for 16 h. Enzyme activity and protein concentration of the crude extract, flow-through, and pure enzyme were determined using the enzyme activity assay and Bradford protein assay (Aminian et al. 2013) and the resulting data used for determining purification yield and specific activity of recombinant ChOA.

### Kinetic characterization of purified ChOA

The optimum pH for the recombinant enzyme activity was determined by the enzyme activity assay at 30 °C under various pH (3–11) conditions. The buffer systems were prepared according to Doukyu et al. (Doukyu et al. 2008). The recombinant ChOA activity was also assayed at different temperatures (30 °C–80 °C) in order to determine the recombinant enzyme optimum thermal activity. The K_m_ and V_max_ values for cholesterol were estimated from Lineweaver–Burk plots of data obtained with the assay solution containing 0–1 mM cholesterol.

## Results

### Optimization of recombinant ChOA expression

#### Optimal host strain for ChOA expression

**Fig. 1:**
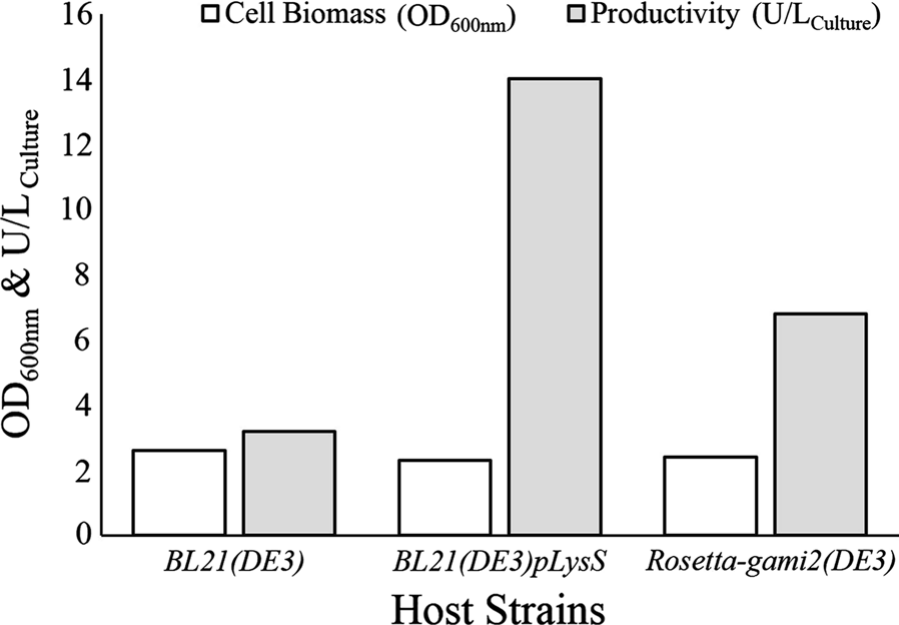
Influence of various hosts on the total productivity of recombinant ChOA and cell biomass production after 6 h post-induction incubation at 37 °C

Evaluation of the *E. coli* host strains productivity for producing recombinant ChOA was performed by transformation of pET24-*ChOA* plasmid into *BL21(DE3)*, *BL21(DE3)pLysS*, and *Rosetta*-*gami2(DE3)*. The host strains were simultaneously induced with 0.5 mM IPTG and the protein expression continued for 6 h at 37 °C, 160 rpm. Following sonication, the cell lysates were centrifuged to remove insoluble materials, and the resulting supernatants were collected to determine the units of enzyme produced per liter of the culture media. The *BL21(DE3)pLysS* cells yielded the maximum level of active recombinant ChOA with 14 U/L activity (Fig. 1). In addition, the total amount of active enzyme obtained from *Rosetta*-*gami2(DE3)* (6.8 U/L) was higher than that of *BL21(DE3)* (3.2 U/L).

#### Optimal culture media for ChOA expression

**Fig. 2:**
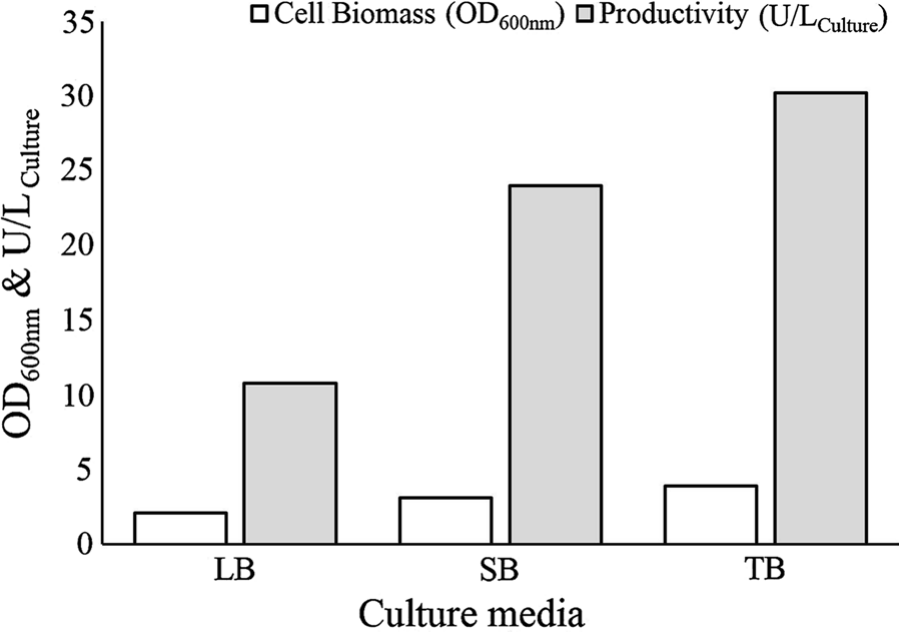
ChOA productivity and cell biomass accumulation in various culture media

To achieve the optimum production of soluble ChOA in *BL21(DE3)pLysS*, three different culture media (LB, TB, and SB) were evaluated. To compare the effect of the different culture media, overnight culture of *BL21(DE3)pLysS* containing pET24-*ChOA* was developed in LB at 37 °C. Pre-culture inoculum (1%) was transformed into freshly prepared LB, TB, and SB media and incubated at 37 °C until OD_600nm_ reached 0.6. Subsequently, the cultures were induced with 0.5 mM IPTG and were grown for another 6 h at 37 °C, 160 rpm. Comparison of cell density and total enzyme activity in different media is demonstrated in Fig. 2. The highest biomass accumulation (OD_600nm_ = 3.9) was achieved by TB medium. Also, ChOA assay indicated that recombinant protein productivity increased in TB medium when compared to the other media.

#### Pre-induction growth optimization

**Fig. 3:**
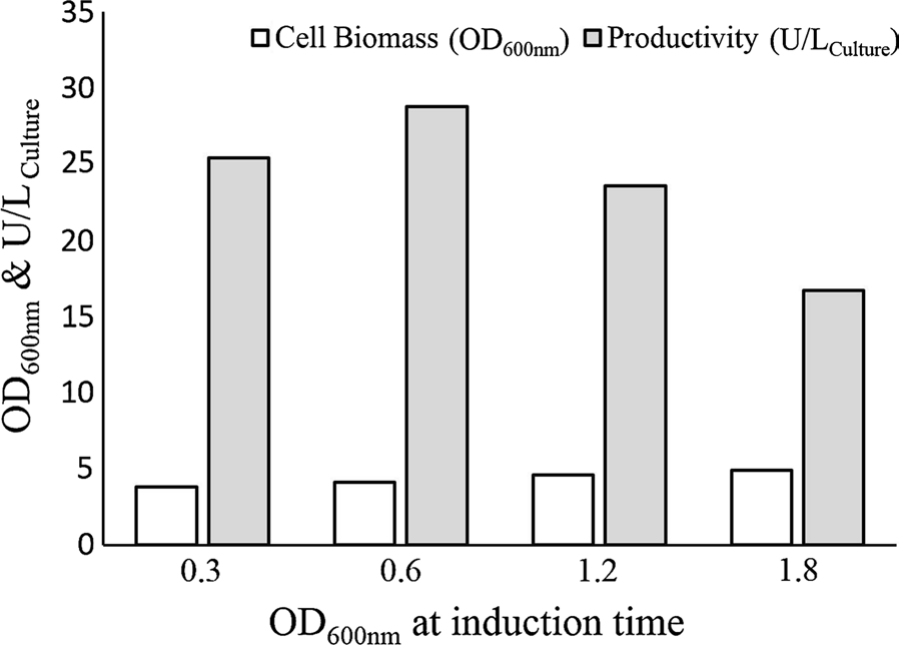
Effect of pre-induction growth on the final growth rate and ChOA expression. Protein expression was induced at various bacterial growth phases (0.3, 0.6, 1.2, and 1.8)

In order to determine the optimum induction time, four shake flasks were examined in parallel, with each culture induced at different growth phases. Cultures were induced with 0.5 mM IPTG when the OD_600nm_ reached 0.3, 0.6, 1.2, and 1.8, representing early exponential, mid-exponential, late exponential, and stationary phases, respectively. The results, depicted in Fig. 3, indicate that ChOA yield was maximized to 28.8 U/L when induction was made at the mid-exponential growth phase (OD_600nm_ = 0.6).

#### Inducer concentration optimization

**Fig. 4:**
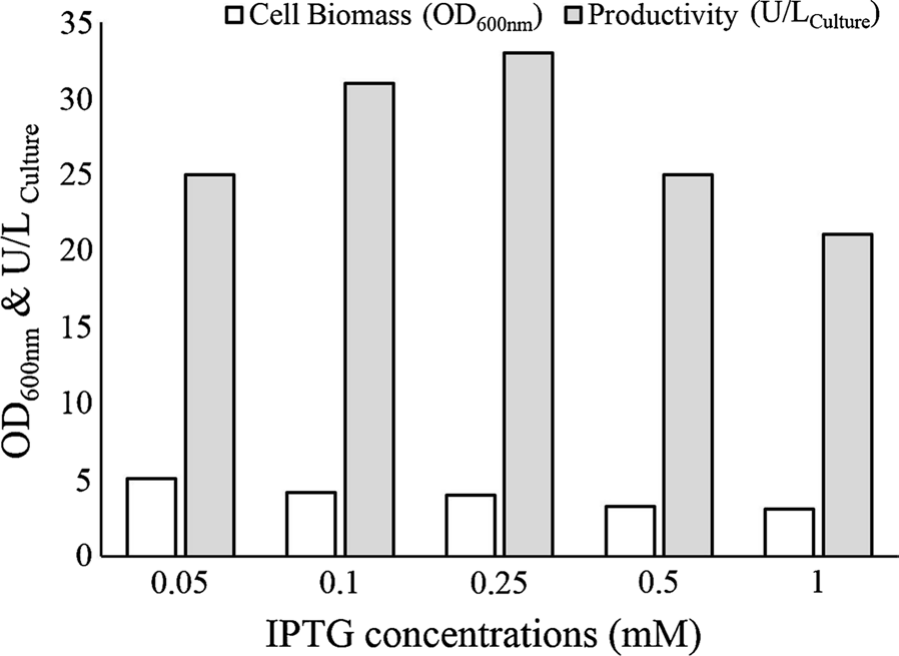
ChOA activity levels and cell growth at various IPTG concentrations (0.05, 0.1, 0.25, 0.5, and 1 mM) after 6 h incubation at 37 °C

Further, the effect of IPTG concentrations (0.05, 0.1, 0.25, 0.5, 1 mM) on ChOA productivity was investigated under the best conditions achieved so far (*BL21(DE3)pLysS*, TB medium, induction at OD_600nm_ = 0.6). Figure 4 presents the results obtained by the performed experiments. As IPTG gradually increased up to 0.25 mM, the productivity also increased in the same way. Nevertheless, beyond 0.25 mM, reduction in the active enzyme yield was observed.

#### Optimal induction temperature and post-induction incubation time

**Fig. 5:**
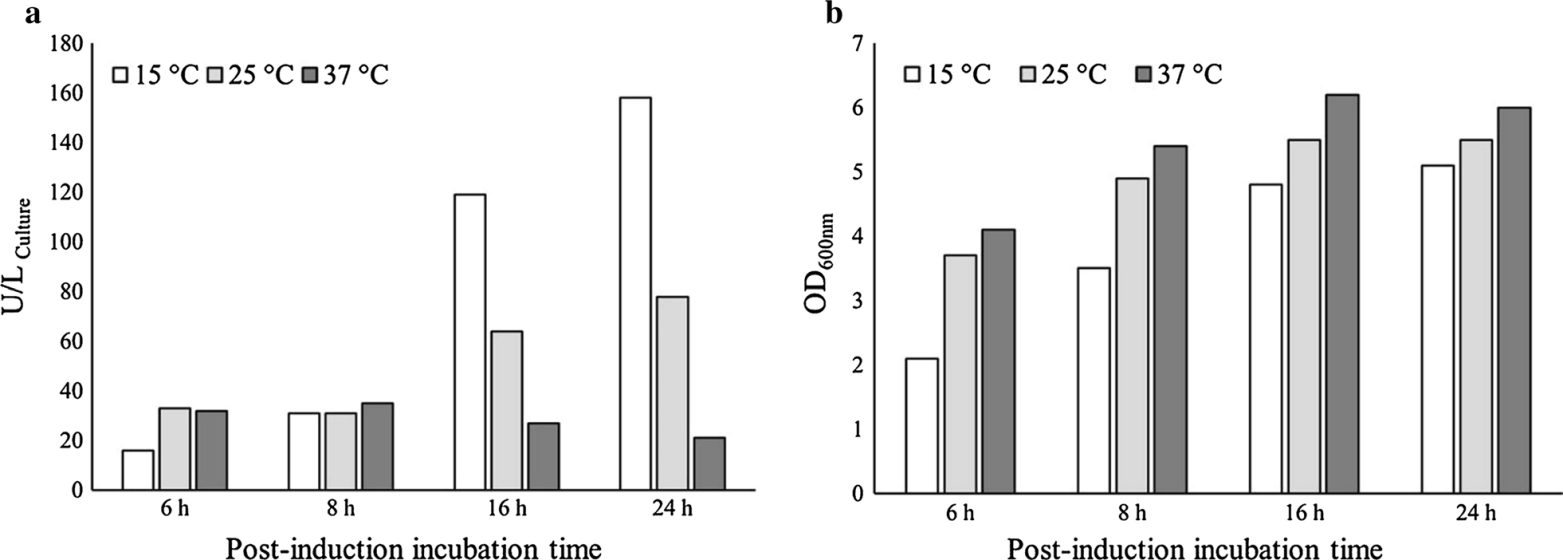
Comparison of ChOA production (**a**) and the cell growth (**b**) at various temperature and post-induction incubation times

To determine the optimal induction temperature and post-induction incubation time, three flasks containing TB media were cultivated under previously optimized conditions. After addition of IPTG (0.25 mM), the flasks were incubated at 15 °C, 25 °C, and 37 °C, separately. During incubation, 2 mL of culture was withdrawn from each flask at different time intervals (6, 8, 16, and 24 h). Enzyme activity assay revealed that recombinant ChOA production was markedly increased considerably when the induced culture medium was incubated at 15 °C for 24 h. As summarized in Fig. 5, the cell density and total enzyme activity decreased when the cultures were incubated at 37 °C even for 16 or 24 h.

### Large-scale enzyme production

All the results above were obtained from small-scale cultures. Collecting all the optimal conditions together, we performed 500 mL culture of *BL21(DE3)pLysS*-pET24-*ChOA* at 15 °C for 24 h by induction with 0.25 mM IPTG at the mid-exponential phase (OD_600nm_ ≃ 0.6). After sonication and obtaining a clarified crude extract by centrifugation, the total enzyme activity and total protein concentration were measured. As shown in Table 1, the total enzyme activity and total protein content were 78.5 U and 63 mg, respectively.

**Table 1:** Summary of the purification procedure for the recombinant choA

Steps	Total activity^a^ (U)	Total protein (mg)	Specific activity (U/mg)	Purification (fold)	Yield (%)
Crude extract^b^	78.5	63	1.25	1	100
Ni-CAM affinity chromatography	67.7	9.57	7.07	5.66	86.2

^a^Cholesterol oxidation activity was assayed by measuring H_2_O_2_ generation

^b^Crude extract was obtained from 500 mL of the culture of *BL21(DE3)pLysS*-pET24-*ChoA*

### Purification of recombinant ChOA

**Fig. 6:**
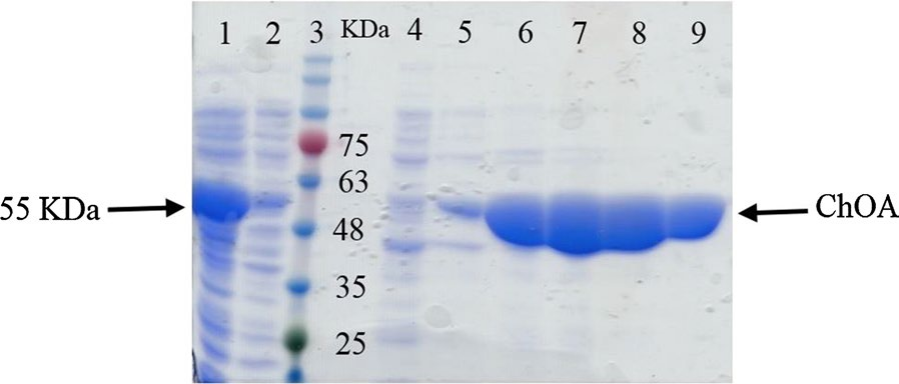
SDS-PAGE analysis of recombinant ChOA purification steps. Lane 1: crude extract, lane 2: column flow-through, lane 3: protein marker, lane 4: 50 mM imidazole elution, lanes 5–9: 200 mM imidazole elution

The recombinant ChOA protein containing N-terminal 6 × His-Tag was purified by nickel column affinity chromatography. The pre-column, flow-through, and eluted fractions were analyzed by SDS-PAGE, with the results indicating that pure ChOA was efficiently eluted by 200 mM imidazole. As displayed in Fig. 6, lanes 5–9, ChOA was highly purified. Eluted fractions containing pure ChOA were pooled and dialyzed against 50 mM of sodium phosphate buffer at pH 7. Table 1 summarizes the data of purification steps. The overall yield of 86% and the approximately 5.7-fold increase in the overall purification were achieved by Ni-CAM affinity chromatography.

### Properties of the purified cholesterol oxidase

**Fig. 7:**
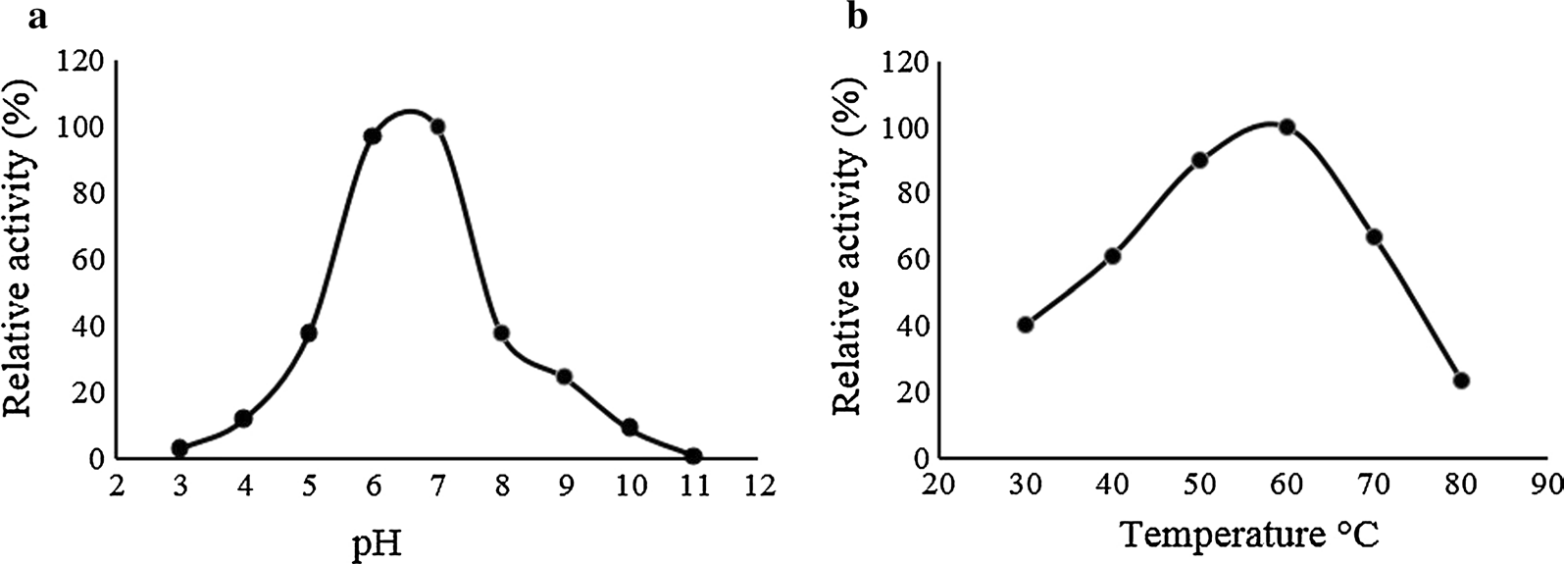
Effect of different pH and temperatures on the activity of the recombinant ChOA. **a** Effect of pH: The residual activity was examined by monitoring H_2_O_2_ generation at 25 °C. The buffer systems (0.1 M) utilized were glycine–HCl (pH 3.0), citrate-sodium citrate (pH 4.0), CH_3_COOH–CH_3_COONa (pH 5.0), NaH_2_PO_4_–Na_2_HPO_4_ (pH 6.0), Tris–HCl (pH 7.0–9.0), and Na_2_CO_3_–NaHCO_3_ (pH 10.0–11.0). **b** Effect of temperature: enzyme activity was assayed in 0.1 mM potassium phosphate buffer pH 7.0 at the indicated temperatures

**Fig. 8:**
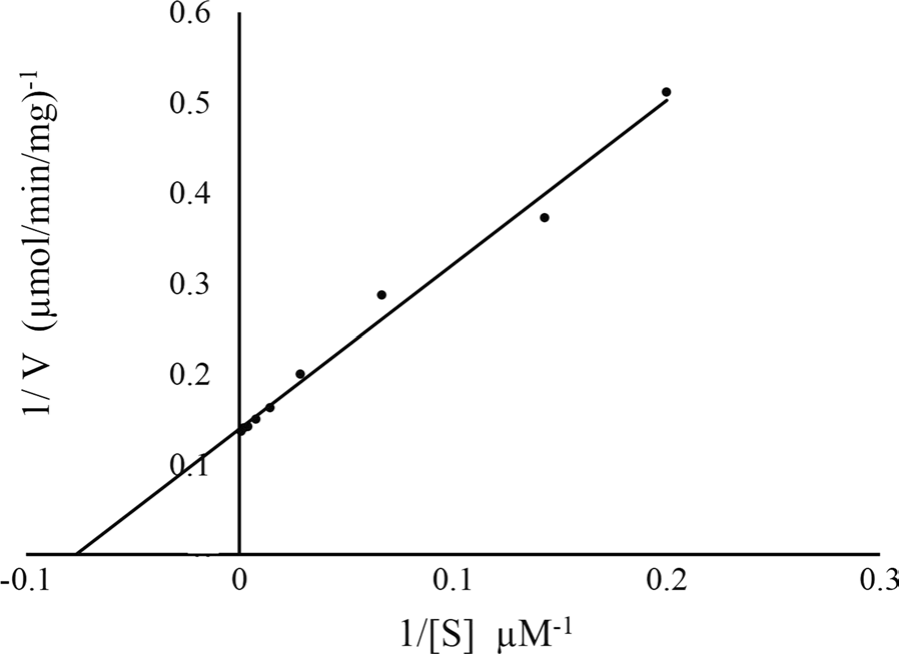
Lineweaver–Burk plot for cholesterol oxidase in the presence of various concentrations of cholesterol (0–1 mM) as substrate

The ChOA activity was measured at different values of pH and temperature. The enzyme activity retained more than 95% of its maximal activity within the pH range of 6–7 at 25 °C (Fig. 7a). The optimum temperature for the ChOA activity was determined, with the results indicating that the optimal temperature for ChOA activity was 60 °C (Fig. 7b). Further, the enzyme retained more than 60% of its activity at the temperatures from 40 °C to 70 °C under the test conditions. In addition, the enzyme had a relatively low activity at 30 °C (42%) and 80 °C (24%) compared to 60 °C. To calculate the K_m_ and V_max_ values of purified ChOA, the activity of the enzyme was assayed with a range of cholesterol concentrations (0–1 mM) at 25 °C, 0.1 M of potassium phosphate buffer pH 7. For K_m_ and V_max_ estimation, 1/V was plotted against 1/[S] in a Lineweaver–Burk plot (Fig. 8). Results indicated that the K_m_ and V_max_ values were found to be 13 µM and 7.2 µmol min^−1^ mg^−1^ respectively.

## Discussion

Cholesterol oxidase as a bacterial flavoenzyme has a great commercial value with a wide range of applications in various fields (Kumari and Kanwar 2012). In light of this, the most efficient production of the enzyme is desired in a recombinant form. There are several obstacles against the heterologous protein expression which results in the production of a recombinant protein at a very low or zero level. One of the simplest ways to address these issues is selecting a suitable host strain and optimizing the expression conditions (Rosano and Ceccarelli 2014). In the current study, several parameters were selected for optimization of the cholesterol oxidase production.

In the first step, three different *E. coli* hosts were used to produce recombinant ChOA. Among them, *BL21(DE3)pLysS* expressed relatively high levels of the active enzyme. pET expression system based on T7 promoter was used for efficient expression of our desired gene. High transcription rate is the advantage of this system but in some cases, this can lead to accumulation of misfolded proteins in inclusion body due to saturation of protein folding machinery (Bahreini et al. 2014). *BL21(DE3)pLysS* was designed to resolve this problem. In this way, pLyS plasmid consistently produces phage T7 lysozyme which can bind to T7 RNA polymerase and partially prevents the transcription of the recombinant gene that is under the control of T7 promoter (Stano and Patel 2004).

Culture media should be accurately selected given their effect on cell growth and metabolism. Therefore, the yield of protein expression may be affected by culture media composition (Sivashanmugam et al. 2009). In this regard, we performed our experiments using three different media consisting of LB, SB, and TB. We found that cholesterol oxidase productivity in TB media increased approximately by three times in comparison with LB media. High concentrations of yeast extract, superior buffering capacity, and the use of glycerol as the carbon source supplement enable high biomass accumulation and high ChOA production (Collins et al. 2013).

Bacterial growth phase at the time of induction as well as inducer concentration also affect the production of recombinant proteins (Ahmad et al. 2018). Accordingly, the effects of these parameters on our target protein yield were next examined individually. Figure 3 indicates that the productivity of the enzyme did not change significantly when IPTG was added during the entire exponential phase. However, the expression level decreased when induction was made at the stationary growth phase. Evaluation of biomass production during different induction times revealed that the addition of IPTG at the early exponential phase reduced biomass production; in return IPTG addition at the stationary phase led to increased biomass accumulation. When induction was made at the early exponential growth phase, the bacterial metabolic resources were channeled to producing recombinant protein constituting 50% of the total cellular protein (Jevševar et al. 2005; Jin et al. 2012). Based on this reasoning, we should expect lowered cellular growth rate following the early exponential phase induction. Our experiment also showed that great production of recombinant ChOA was obtained when IPTG concentration was 0.25 mM.

Several studies have suggested that post-induction temperature as well as incubation time can affect the activity and yield of recombinant protein production (Caspeta et al. 2009; Khow and Suntrarachun 2012; Sahdev et al. 2008; Saïda 2007). In addition, Mizukami et al. have reported that different expression temperatures finally led to equal-mass production of the recombinant enzyme with different total activity. They suggested that in the cells cultured at a lower temperature the recombinant enzyme seems to exist as an active form, while as a rather denatured form in the cells cultured at a higher temperature (Mizukami et al. 1986). In light of these findings, we also investigated the effect of different post-induction temperatures (15 °C, 25 °C, and 37 °C) along with post-induction incubation times (6, 8, 16, and 24) on the yield of recombinant ChOA. As can be seen clearly in Fig. 5, reducing temperature down to 15 °C together with extending the incubation period up to 24 h enhanced the enzyme productivity by approximately 7.5 times relative to the same condition at 37 °C. Generally, metabolic burden usually occurs in recombinant bacteria (Bentley et al. 1990). Accordingly, high-rate produced recombinant proteins may accumulate in insoluble aggregates (inclusion body) as a direct consequence of overwhelming the host folding machinery (Sørensen and Mortensen 2005). In addition, hydrophobic interactions which are a key factor in the formation of inclusion bodies would decline if temperature is lowered (Kiefhaber et al. 1991; Löw et al. 2012; Ma et al. 2013).

Furthermore, in order to study the enzymatic characteristics of the recombinant ChOA, large-scale production of ChOA was performed under the optimized conditions. Maximum yield of recombinant ChOA production was determined to be 1.25 U/mg. Nomura et al. produced ChOA by *Streptomyces* sp. SA-COO and *E. coli* JM109. They achieved 0.69 U/mg ChOA when cholesterol oxidase was produced by *Streptomyces* sp. SA-COO. Further, they obtained 1.5 U/mg recombinant enzyme when N-terminal modified *ChOA* was expressed in *E. coli* JM109. The characterization of purified recombinant ChOA indicated that the recombinant enzyme was most active at 50 °C–70 °C, with 60 °C being the optimum temperature, which is the same as that of other *Streptomycetes* (Lartillot and Kedziora 1990; Nishiya et al. 1997; Tabatabaei Yazdi et al. 2001; Tomioka et al. 1976). However, the enzyme retained only 24% of its activity at 80 °C. Furthermore, activity assay at different pH values revealed that the optimum pH for enzyme activity was 7. Most reports have demonstrated the optimum pH for cholesterol oxidase from other *Streptomycetes* as about 6.5–8 (Kamei et al. 1978; Lartillot and Kedztora 1990; Smith and Brooks 1976). The K_m_ value for cholesterol was calculated to be 13 µM for purified ChOA. This value is consistent with the study of Nishiya et al. (Nishiya et al. 1997), which is lower than that of the enzymes from *S. hygroscopicus* and *S. virginiae* (Gadda et al. 1997; Li et al. 2010).

In conclusion, the results of our study suggested that optimization of ChOA expression conditions in *E. coli* significantly enhanced the enzyme productivity by approximately 50 times. The affinity purified ChOA retained the enzyme characteristics as reported previously.
